# Optimization of Plasma Sample Pretreatment for Quantitative Analysis Using iTRAQ Labeling and LC-MALDI-TOF/TOF

**DOI:** 10.1371/journal.pone.0101694

**Published:** 2014-07-02

**Authors:** Magdalena Luczak, Lukasz Marczak, Maciej Stobiecki

**Affiliations:** Institute of Bioorganic Chemistry, Polish Academy of Sciences, Poznan, Poland; Moffitt Cancer Center, United States of America

## Abstract

Shotgun proteomic methods involving iTRAQ (isobaric tags for relative and absolute quantitation) peptide labeling facilitate quantitative analyses of proteomes and searches for useful biomarkers. However, the plasma proteome's complexity and the highly dynamic plasma protein concentration range limit the ability of conventional approaches to analyze and identify a large number of proteins, including useful biomarkers. The goal of this paper is to elucidate the best approach for plasma sample pretreatment for MS- and iTRAQ-based analyses. Here, we systematically compared four approaches, which include centrifugal ultrafiltration, SCX chromatography with fractionation, affinity depletion, and plasma without fractionation, to reduce plasma sample complexity. We generated an optimized protocol for quantitative protein analysis using iTRAQ reagents and an UltrafleXtreme (Bruker Daltonics) MALDI TOF/TOF mass spectrometer. Moreover, we used a simple, rapid, efficient, but inexpensive sample pretreatment technique that generated an optimal opportunity for biomarker discovery. We discuss the results from the four sample pretreatment approaches and conclude that SCX chromatography without affinity depletion is the best plasma sample preparation pretreatment method for proteome analysis. Using this technique, we identified 1,780 unique proteins, including 1,427 that were quantified by iTRAQ with high reproducibility and accuracy.

## Introduction

Shotgun proteomic quantification using isobaric tags is one of the most effective methods for analyzing changes in plasma proteomes of diseased cells and tissues [1], [2]. Tandem mass tags (TMT) and isobaric tags for relative and absolute quantitation (iTRAQ) techniques are ideally designed for non-targeted biomarker discovery. The advantage of iTRAQ over TMT is its ability to perform relative quantification of up to eight treatments in a single liquid chromatography-tandem mass spectrometry (LC-MS/MS) analysis. In the iTRAQ approach, peptides from different biological samples are first covalently bound to a set of different chemical “tags” with identical masses (*i.e*., isobaric tags) so that a mixture of labeled peptides can then be identified through MS/MS. The relative peptide abundance can be quantified by measuring the relative abundances of the individual isobaric tags. Thereafter, the identified potential biomarkers must be validated and precisely measured using a targeted method, such as selected/multiple reaction monitoring (SRM/MRM) [3], [4] or parallel reaction monitoring (PRM) [5], [6]. The method also has a few drawbacks. The iTRAQ reagents are expensive, especially compared to label-free methods. However, the ability to multiplex up to eight different samples significantly reduces the cost of analysis. In addition, the method requires certain tandem mass spectrometers equipped with collision-induced dissociation (CID), pulsed Q collision induced dissociation (PQD), higher-energy C-trap dissociation (HCD), and electron transfer dissociation (ETD). Recent reports have also shown the phenomenon of “ratio compression” [7]. Too wide an isolation window used for selection and subsequent fragmentation of ions can influence the accuracy of the labeled fragments and the quantification ratio [7]. However, this potential problem has not disrupted this method's enhanced popularity in biomarker research [8], [9].

Human blood plasma is one of the most studied biological fluids and is the main type of sample used for disease diagnosis. Blood perfusion through different organs and tissues can change the plasma composition and modify existing proteins, which may vary with specific conditions. The plasma proteome can be correlated with specific physiological or pathological states. However, the proteomic analysis of plasma is analytically challenging due to its highly dynamic constituent protein concentration range (greater than 10 orders of magnitude) [10]. Additionally, the 10 most abundant proteins comprise approximately 90% of the total plasma protein mass [11]. The most common approach used to facilitate the proteomic analysis of plasma is to reduce the complexity by fractionating the sample. Several studies on the efficiency and reproducibility of different affinity-depletion platforms have been published [12], [13]. Centrifugal ultrafiltration has been extensively used to remove high-molecular-weight species from plasma. Filters with different cut-offs ranging from 10 to 30 and 50 kDa have been used for biomarker discovery in diseases, such as ovarian cancer [14] and hepatocellular carcinoma [15]. This simple method enriches the sample in low-molecular-weight protein fractions, which are considered an important source for biomarkers [11].

Recent reviews by Hoffman et al. [16] and Pernelmann et al. [17] describe the increasing number of complex approaches that have been developed. Unfortunately, single-step protocols have been used to identify only 100–200 plasma proteins. However, complex, multi-step protocols, which can identify more than 2,000 proteins, are often time-consuming and involve expensive reagents or equipment. This dilemma has been previously noted [18]-[20].

Because the likelihood of finding a new biomarker increases with the number of proteins profiled, the aim of this study was to determine the best pretreatment method to comprehensively profile the human plasma proteome. Our goal was to optimize the known approaches to identify a high number of proteins in a relatively inexpensive, simple, and rapid way.

The first step of this study involved optimizing plasma sample preparation to yield the best overall results for the number of identified proteins through matrix-assisted laser desorption/ionization-time of flight/time of flight (MALDI-TOF/TOF) analyses. Qualitative analysis of the results was performed. The second step involved selecting the techniques that yielded the highest number of identified proteins and using these techniques for plasma quantification through iTRAQ labeling. To the best of our knowledge, this study is the first report to compare centrifugal ultrafiltration, SCX chromatography with fractionation, and affinity depletion in a single experiment to analyze the plasma proteome. Moreover, this is the only study on iTRAQ-labeled peptides using the new UltrafleXtreme (Bruker Daltonics) MALDI-TOF/TOF mass spectrometer.

## Materials and Methods

### 1. Plasma samples

The study protocol conformed to the ethical guidelines of the World Medical Association Declaration of Helsinki. Before beginning this project, the appropriate approval was obtained from the Bioethical Commission of Karol Marcinkowski University of Medical Sciences (no. 34/06 05.04.2006). Human blood samples from 35 healthy donors, who provided written informed consent, were harvested in collection tubes with EDTA and prepared as described previously [21]. To minimize plasma diversity, samples were collected and prepared by one person, during one day, under identical conditions. After centrifugation at 1,000×*g* for 10 min, the plasma was separated from the blood cells. The plasma samples were then centrifuged at 16,000×*g* for 15 min at 4 °C, and the supernatants were frozen at −80°C until use.

### 2. Sample preparation

#### 2.1. Procedures without iTRAQ labeling

In all of the sample preparation approaches, the protein concentrations were estimated using a Nanodrop spectrophotometer (Thermo Scientific) to obtain the same protein quantities in each analysis. Protein mixtures from the different fractionation procedures were aliquoted into 10 µg samples and prepared for in-solution digestion. All experiments (sample preparation, digestion, and MS analyses) were repeated four times.

#### 2.1.1. Plasma without fractionation (WF)

Two microliters of raw plasma was diluted with 59 µl of Milli-Q water in a 0.22 µm spin filter tube (Agilent Technologies, USA) and spun at 14,000×*g* in a centrifuge (Micro 220R, Hettich, Germany) for 1 min. The diluted plasma samples were aliquoted into 10 µg samples and prepared for in-solution digestion.

#### 2.1.2. Immunoaffinity depletion (MARS)

Plasma samples were processed to decrease plasma complexity *via* depleting highly abundant proteins using a MARS-Hu7 affinity column (**M**ultiple **A**ffinity **R**emoval **S**ystem- **Hu**man **7**; Agilent Technologies, USA). MARS Hu7 spin columns remove the seven most abundant proteins in plasma (albumin, IgG, α-1-antitrypsin, IgA, transferrin, haptoglobin, and fibrinogen). Human plasma (20 µl) was diluted to 400 µl with “Buffer A” (Agilent Technologies) and spun twice at 14,000×*g* for 1 min in a 0.22 µm spin filter tube (Agilent Technologies, USA). Two hundred microliters of diluted plasma sample were added to a MARS column and centrifuged 30 sec at 200×*g*. Next, 400 µl “Buffer A” was added to the column and centrifuged 1 min at 200 x g. The F1 flow-through fraction was collected. Four hundred microliters of “Buffer A” was added to the column and then centrifuged 1 min at 200×*g*. The F2 flow-through fraction was collected and combined with the F1 fraction. “Buffer B” (Agilent Technologies) (2 ml) was used to elute the bound proteins. The cartridge was re-equilibrated for the next sample with “Buffer A”. Aliquots from the combined F1 and F2 flow-through fractions (FT) containing low-abundance proteins and the bound fractions (B) containing the seven highly abundant proteins were desalted three times through buffer exchange using 50 mM NH4HCO3 and a centrifugal filter with a 5-kDa cutoff (Amicon Ultra, Millipore). The protein mixtures were aliquoted into 10 µg samples and prepared for in-solution digestion.

#### 2.1.3. Ultrafiltration depletion (Ami50)

Amicon Ultra-4 50K filters (Millipore, Ireland) were rinsed in deionized Milli-Q water. Fifty microliters of plasma was diluted with 450 µl of 20% (v/v) ACN in 50 mM NH4HCO3, incubated at 95°C for 5 min in a 0.22 µm spin filter tube (Agilent Technologies, USA), and spun at 14,000×*g* in a centrifuge for 1 min. The samples were then centrifuged at 3,500×*g* in the Amicon filters for 30 min. The filtrates were evaporated in a vacuum centrifuge (CentriVap, Labconco) to approximately 50 µl. The plasma protein mixtures were aliquoted into 10 µg samples and prepared for in-solution digestion.

#### 2.1.4. Strong cation exchange chromatography (SCX)

Two microliters of raw plasma was diluted with 59 µl of Milli-Q water in a 0.22 µm spin filter tube (Agilent Technologies, USA) and spun at 14,000×*g* in a centrifuge for 1 min. The diluted plasma was aliquoted into eight 10 µg samples and prepared for in-solution digestion.

After digestion, the peptides were fractionated using two different SCX systems, including a cartridge system (AB Sciex) and a spin system (Microspin; The Nest Group, Inc.), according to the manufacturer's instructions. The peptides were sequentially eluted from the columns with increasing KCl concentrations. Four fractions were collected in 100, 200, 350, and 500 mM KCl. Because the samples were fractionated into four fractions, the peptides from four pooled 10-µg samples were used for the SCX approach.

#### 2.1.5. In-solution digestion

Ten-microgram aliquots of plasma proteins prepared using the four approaches described above (WF, MARS, Ami50, and SCX) were diluted with 15 µl of 50 mM NH4HCO3 and reduced with 5.6 mM DTT for 5 min at 95°C. The samples were then alkylated with 5 mM iodoacetamide for 20 min in the dark at RT. The proteins were digested with 0.2 µg of sequencing-grade trypsin (Promega) overnight at 37°C.

#### 2.2. Procedures with iTRAQ labeling

Two of the four approaches described above, MARS and SCX, were selected for the iTRAQ experiments. For these experiments, the plasma protein mixtures were aliquoted into 50- and 100-µg samples according to the manufacturer's instructions. In the MARS approach, the plasma samples were processed through affinity depletion, and the flow-through fractions (FT) were desalted and concentrated as indicated in section 2.1.2, and then aliquoted into 50- and 100 µg samples. For the SCX fractionation experiments, 5 µl of raw plasma was diluted with 45 µl of Milli-Q water and then aliquoted into 50- and 100 µg samples. The prepared plasma protein samples were then processed using the iTRAQ Reagents Application Kit – Plasma (AB Sciex). All subsequent solutions were included in the iTRAQ reagent kits. The plasma proteins (50 and 100 µg) from the MARS and SCX pretreatments were suspended in dissolution buffer, denatured, reduced, and alkylated. TPCK-treated trypsin (AB Sciex) was reconstituted at 1 µg/µl in Milli-Q water, and 10 µg of trypsin was added to each sample. Digestion was performed overnight at 37°C. After digestion, the peptides were labeled with the iTRAQ labeling reagents 114, 115, and 117. Because we used labeling to identify differences in the compound concentrations in real samples, we mimicked real conditions by varying the protein sample concentrations. The samples were labeled as follows: 115 and 117–50 or 100 µg of plasma proteins from the SCX approach and 114 and 117–50 or 100 µg of plasma proteins from the MARS approach. Therefore, the predicted median values were 1∶2 for the 115/114 and 117/114 peak area ratios. The median values were then calculated from the reporter peak area ratios of all labeled peptides for a given protein. This procedure also allowed us to examine the labeling efficiency for the same peptides at different concentrations. Labeling was performed for 1 h at room temperature, and after incubation, the reaction was quenched with 100 µl of 0.1% TFA. The labeled peptides from each approach were combined into one tube, diluted, and cleaned using an SCX cartridge system (AB Sciex) to fractionate and remove substances that might interfere with the LC-MS/MS analysis. The peptides from the MARS and SCX approaches were sequentially eluted from the column using 100, 200, 350, and 500 mM KCl in 10 mM KH2PO4/25% ACN, pH 3.0 and evaporated for 15 min in a vacuum centrifuge (CentriVap, Labconco).

### 3. Mass spectrometry analysis

#### 3.1. Off-line LC-MS/MS analysis

The samples prepared from each approach, with and without iTRAQ labeling, were subjected to nano-LC separation using an EASY-nLC Proxeon (Bruker Daltonics, Germany) coupled to a Proteineer fc II (Bruker Daltonics, Germany) fraction collector. The samples were separated using a C18 pre-column (Thermo Scientific) connected to a 25 cm-long, 75 µm-i.d. C18 column (Dionex). The samples were separated at 0.3 µl/min using a linear ACN gradient from 0 to 65% for 215 min (solvent A: 0.1% trifluoroacetic acid; solvent B: ACN 0.1% trifluoroacetic acid). Beginning 17 min after the analytical gradient was initiated, the LC fractions were automatically mixed with an α-cyano-4-hydroxycinnamic acid (HCCA) MALDI matrix solution (1.5 mg/ml HCCA in 93% ACN, 0.1% TFA, 1 mM NH_4_H_2_PO_4_) and deposited onto an MTP AnchorChip 384 target plate. In total, 384 fractions were collected. The LC system and fraction collector were controlled by Bruker's Hystar software (version 3.2 SR2). The MALDI-TOF/TOF (UltrafleXtreme, Bruker Daltonics) instrument was operated in the positive ion mode and controlled by the Compass for Flex software, version 1.3 (FlexControl 3.3, FlexAnalysis 3.3, Bruker Daltonics); 5,000 laser shots were accumulated per spectrum in the MS and MS/MS modes. The spectrometric analysis was performed in an automatic data-dependent mode. The non-redundant precursor peptides were selected for MS/MS using the WARP-LC 1.3 software (Bruker Daltonics) with a signal-to-noise threshold of 12. The MS spectra were externally calibrated using the Peptide Calibration Standard mixture (Bruker Daltonics). Additionally, the MS/MS spectra were internally calibrated using the 114/117 or 115/117 tags.

#### 3.2. Protein identification and validation

The data were analyzed using the ProteinScape (Bruker Daltonics) database software, and MASCOT 2.3 (Matrix Science, London, UK) was used as a search engine. The search parameters were as follows: carbamidomethylation of cysteine residues; precursor-ion mass tolerance, +/−0.3 Da; and fragment-ion mass tolerance, +/−0.5 Da. For data from the iTRAQ-labeling procedures, the iTRAQ 4-plex (peptide label) modification was also used in the search. The false discovery rate (FDR) for peptide identification was 0.05 in all analyses. The SwissProt and NCBI databases were used for protein identification. To reduce false-positive protein identifications, only proteins identified in both databases were considered significant hits. The plasma proteome database, available for free at http://www.plasmaproteomedatabase.org, was used for functional annotation of all identified proteins [22]. All comparisons and compiled lists of identified proteins were generated using the ProteinScape software (Bruker Daltonics).

#### 3.3. Quantification of iTRAQ-labeled proteins

Fully automated, quantitative analyses were performed using the WARP-LC 1.3 software. The relative peptide abundance was quantified using the iTRAQ reporter ion peak area ratios and normalized based on the median ratio for each iTRAQ channel across all proteins. The median 114/117 or 115/117 values and CV values (coefficient of variation) were the major parameters used to validate the data. The median values were then calculated from the reporter peak area ratios of all labeled peptides for a given protein. The quantitative data were exported into Excel (Microsoft) for further analysis. The *p*-values for each protein were generated using the two-tailed Student's t-test to compare the values from four experiments. For detailed information on the identified and quantitated proteins, see File S1.

## Results and Discussion

### Comparison of the different protein/peptide fractionation techniques for protein identification

**Figure 1:**
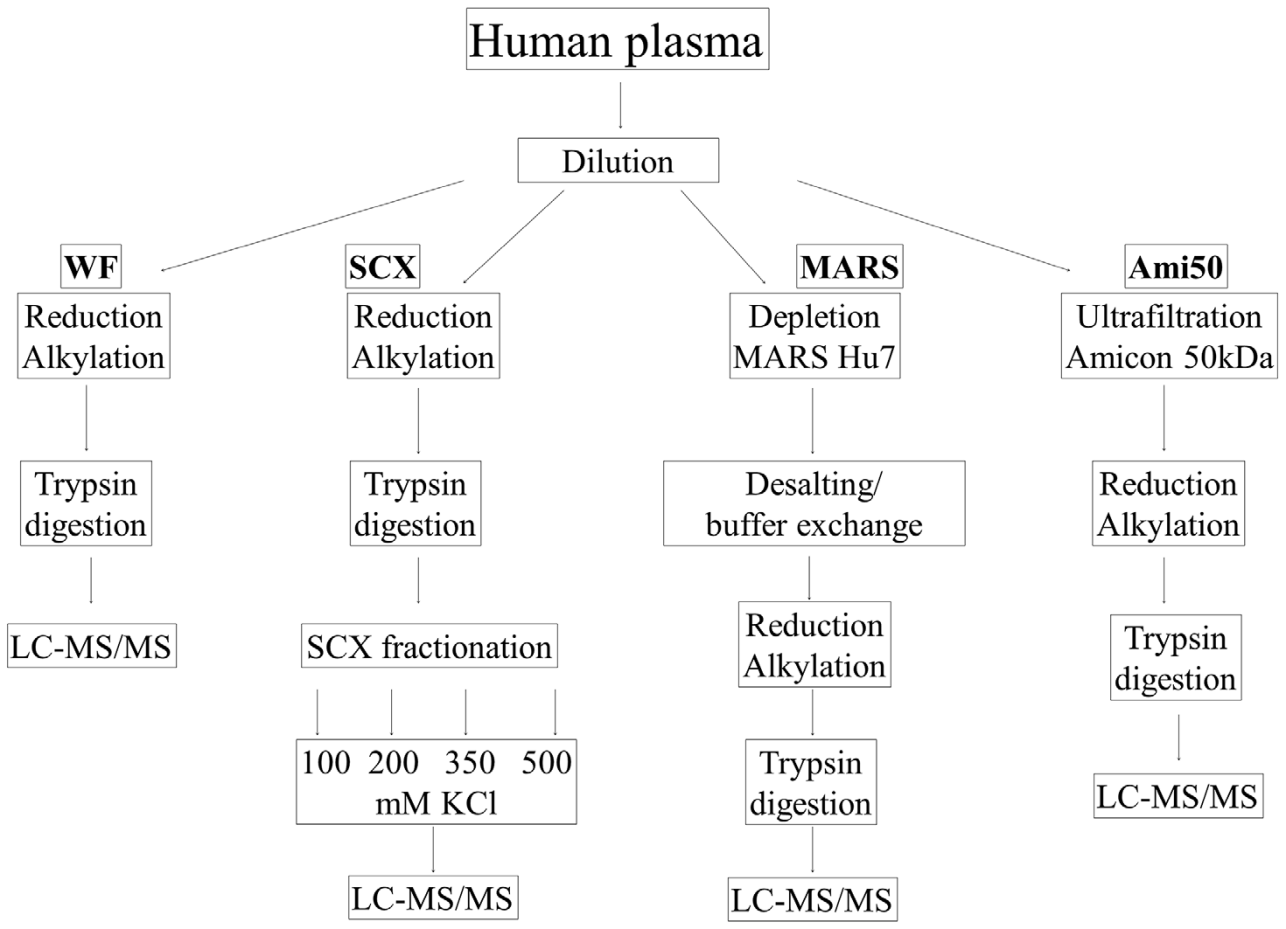
The experimental workflow shows a systematic comparison of the four approaches for plasma sample pretreatment. Centrifugal ultrafiltration (Ami50), SCX chromatography with fractionation (SCX), affinity depletion (MARS), and plasma without any fractionation (WF) were performed to reduce the plasma sample complexity. The peptide mixtures generated were analyzed using LC-MALDI-TOF/TOF.

We propose an optimization procedure that facilitates a comprehensive plasma analysis for the highest number of identified proteins. Because many strategies for plasma depletion are described in the literature, we first evaluated the conditions for sample preparation procedures that yielded the highest number of accurately identified proteins during an analysis. We performed an LC-MALDI-MS/MS analysis of plasma proteins prepared using four different approaches to select the best method, which we defined as the method that yielded the highest number of accurately identified proteins. These methods included centrifugal ultrafiltration, SCX chromatography with fractionation, and affinity depletion, which we compared to plasma without fractionation. The results were qualitatively analyzed. The workflow for the unlabeled experiments is presented in Figure 1. In all cases, a simple and inexpensive in-solution digestion procedure was used. We compared the results with analyses of the plasma without fractionation, plasma centrifuged using Amicon 50 kDa, plasma depleted using MARS-Hu7-, and SCX-fractionated plasma (these approaches are referred to as WF, Ami50, MARS and SCX, respectively). Table 1 shows the number of proteins identified in each approach. To validate the reliability of these measurements, all results were determined in four separate experiments and Student's t-test was used to assess the differences between methods. No significant differences were detected between experiments. Our analyses identified 753, 1098, 1,590 and 1,113 unique proteins from the Ami50, WF, SCX, and MARS approaches, respectively. Combining these techniques, 3,296 unique proteins were identified. However, only 98 proteins were common to all four approaches. The complete list of proteins identified in each approach is reported in Table S1 in File S1.

**Table 1:** The number of identified proteins in four separate experiments with and without iTRAQ labeling.

	Number of nonredundant proteins	Number of unique peptides identified per protein		
Approach	Total	≥3	2	1
**Non-labeled experiment**				
WF	1098±151	148	238	712
Amicon 50 kDa	753±174	99	125	529
MARS FT	1113±297	276	258	579
MARS B	233±51	19	34	180
Cartridge SCX (four fractions)	1590±124	251	323	1016
Spin SCX (four fractions)	1421±131	215	279	927
**iTRAQ experiment**				
MARS FT	1123±269	135	259	729
Cartridge SCX	1780±141	199	405	1176

Each experiment was performed four times, and the number of identified proteins is an average from all experiments. The notes ≥3, 2, and 1 indicate the number of unique peptides per protein.

In the WF approach, undepleted proteins were reduced, alkylated, and digested with trypsin. In this method, 1,098 unique proteins were identified in four separate experiments (Table 1). However, only 148 proteins were identified using 3 unique peptides or more, 238 were identified by 2 peptides, and 712 were single-peptide proteins (Table 1). In the Ami50 approach, ultrafiltration was used to reduce the sample complexity. The plasma proteins were denatured and centrifuged using 50-kDa Amicon filters. As shown recently, many low-molecular-weight (LMW) proteome components in the blood are associated with carrier proteins [23], [24], such as albumin [25]. The fraction of low-molecular-weight proteins and peptides bound to carrier proteins may also comprise an important source for potential disease biomarkers [11], [26]. Previously, we showed that adding ACN to diluted plasma positively affected low-molecular-weight (LMW) protein enrichment [15], [27], [28]. Thus, in the current project, we also used 18% ACN to disrupt any protein/peptide interactions. However, using this approach, only 753 unique plasma proteins were identified in four experiments. Therefore, we concluded that the Ami50 approach was the least useful, perhaps due to the ability of certain proteins to bind filter devices. A spectrophotometric assay of protein concentrations before and after filtration confirmed this hypothesis. Approximately 19% of the initial protein was bound to the microfilter during this step. We expected that using filters with a 50-kDa cutoff would enrich the sample in LMW protein fractions. However, we did not observe high enrichment of LMW proteins in the ultrafiltrate (8.76% of proteins below 30 kDa) compared to the SCX (8.55% proteins below 30 kDa) or MARS FT (5.75% proteins below 30 kDa). Moreover, the top three proteins identified were albumin (MW: 69.3 kDa) with score 5,512.4 and 64 identified peptides, complement factor H (MW: 139 kDa; score: 3,254; 49 peptides), and vitamin D-binding protein (MW: 52.9 kDa; score: 1,872; 15 peptides). These proteins have molecular masses higher than the cutoff of the centrifugal filters used.

**Figure 2:**
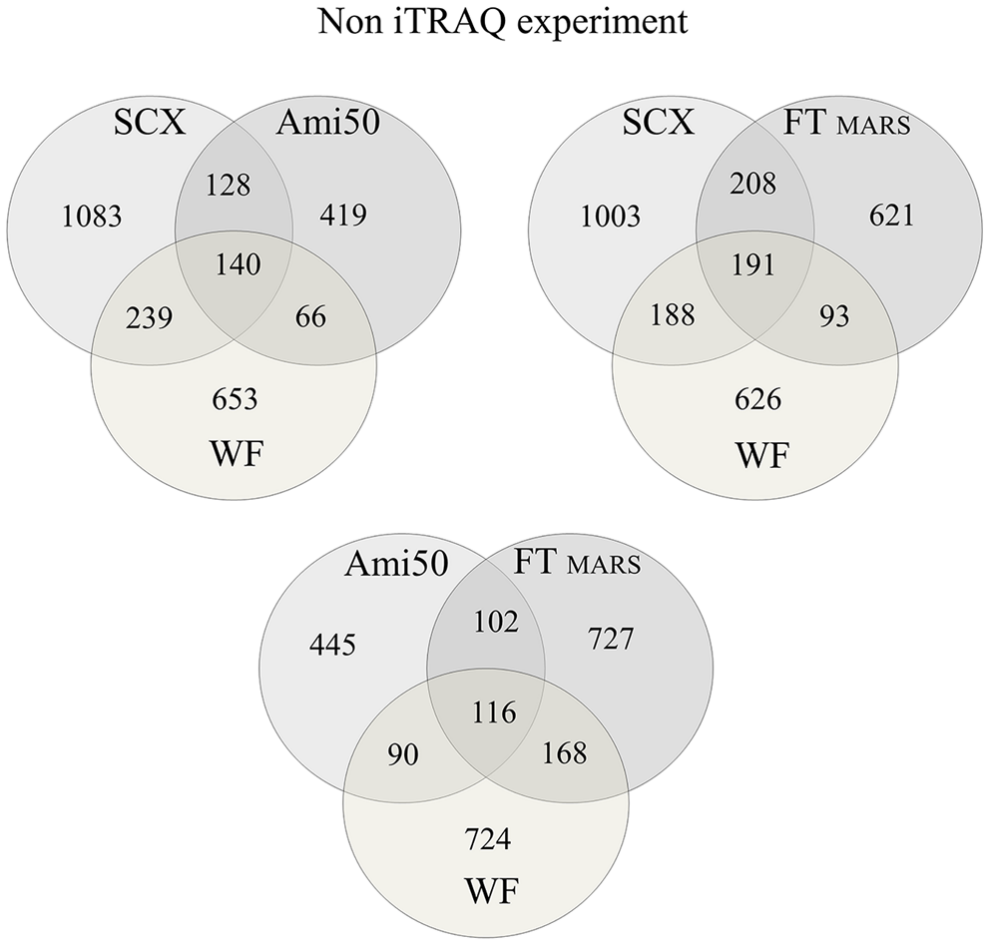
A Venn diagram comparing the results from the WF, Ami50, FT MARS, and SCX approaches in experiments without iTRAQ labeling. The numbers indicate the proteins identified using each approach. A total of 3,296 unique proteins were identified.

The applicability of centrifugal ultrafiltration to studies on LMW protein in plasma has been evaluated in a few previous reports. Greening and Simpson compared four commercially available filter membranes to isolate the LMW component of the human plasma proteome and identified several proteins for the first time; however, the total number of identified proteins was low [29]. Georgiou et al. also reported that ultrafiltration failed to remove albumin and other high-molecular-weight proteins from human plasma [30]. Our results also revealed high-molecular-weight proteins in the ultrafiltrate, including high quantities of albumin and complement factor H (64 and 49 peptides, respectively). In the Georgiou study, ultrafiltration was performed at 12,000×*g*, and, as noted by Tirumalai and co-workers, high-molecular-weight components may pass through the membrane at this high centrifugal force [11]. In addition, undiluted plasma was used, and the ultrafiltration was performed under non-denaturing conditions. Our results were generated using a low centrifugal force (3,500×*g*), 10-fold dilution, and denaturing conditions (18% ACN). Therefore, we compared the results from the WF and Ami50 approaches, which showed only partial overlap of the proteins identified. In total, 419 proteins were identified only with the Ami50 approach, and 653 were identified only using the WF approach, whereas 206 proteins were identified in both samples (Fig. 2). In addition, the MARS FT and SCX approaches were compared with the Ami50 approach and showed a low level of overlap (only 218 and 268 proteins were common to the MARS - Ami50 and SCX - Ami50 methods, respectively). This finding suggests that the Ami50 approach has a certain degree of specificity (i.e., it enriches a specific group of proteins).

**Figure 3:**
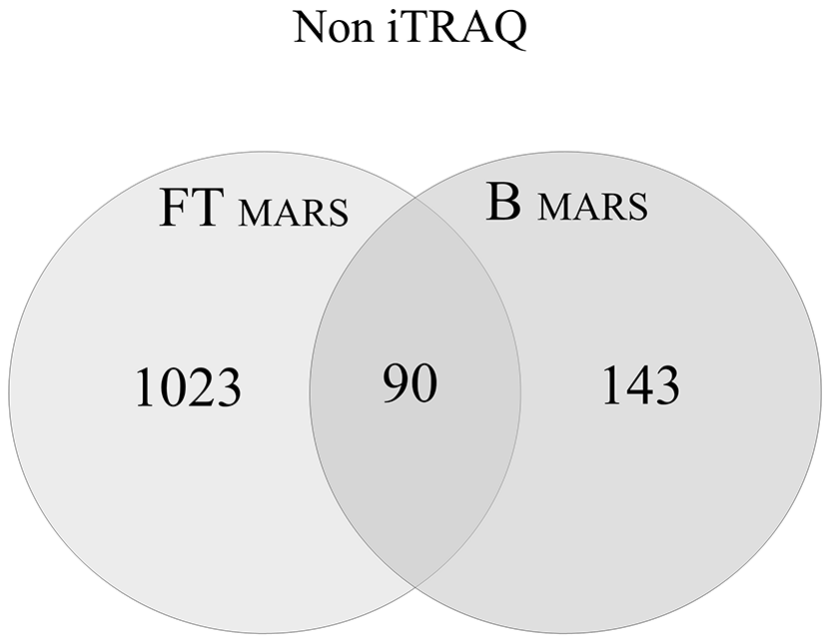
A Venn diagram comparing the results from the FT and B protein fractions after MARS-Hu7 depletion in experiments without iTRAQ labeling. A total of 1,256 unique proteins were identified. Ninety proteins were identified in both fractions, and 1,023 and 143 proteins were unique to the FT and B fractions.

Sample pre-fractionation using affinity chromatography has been shown to improve the detection of low-abundance proteins in plasma [31]. However, one of the potential drawbacks of removing abundant proteins (proteins for which targeted antibodies are present in an affinity column) from plasma is the simultaneous removal of non-targeted proteins. Moreover, in this case, special solvent conditions that disrupt protein interactions cannot be used because the affinity approach requires the proteins to be in their native state. Considering this requirement, we depleted the plasma proteins using a MARS-Hu7 column and then analyzed both fractions, including the flow-through containing non-targeted proteins (FT) and the bound fraction containing the targeted high-abundance proteins (B). We identified 1,113 and 233 unique proteins in FT and B, respectively. In the B fraction, we detected 208 different proteins in addition to the seven targeted proteins, which were identified with high sequence coverage (see Table S1 in File S1). Moreover, all seven proteins that should have been depleted were identified in the FT fraction. In addition, the MARS approach showed the lowest level of reproducibility between experiments (Table 1). Figure 3 shows a comparison of the FT and B results. In total, 90 proteins were identified in both fractions, and 1,023 and 143 proteins were unique to the FT and B fractions, respectively. Our results suggest that analyzing only the FT fraction would significantly decrease the amount of obtained information.

A literature search suggests that the removal of non-targeted proteins should be evaluated in detail. Gundry and co-workers described the ‘albuminome’, which includes 35 plasma proteins that co-elute with albumin using the anti-HSA (human serum albumin) depletion system [23]. Others have also demonstrated non-targeted protein removal using depletion methods; in fact, tens of proteins were identified in the bound fractions from three different affinity systems (MARS-Hu6, MARS-Hu14 and Proteoprep20) [24]. It was concluded that the bound and depleted fractions from an affinity-depleted plasma sample might be useful for biomarker discovery [24]. In fact, albumin, a carrier protein, binds various low-molecular-weight proteins through protein-protein or protein-antibody interactions. Stempfer et al. reported 24 proteins from the bound fraction using MARS-Hu6 [32]. In light of these results and the work presented herein, an analysis of the depleted and bound fractions is necessary.

In the SCX approach, plasma proteins without depletion were reduced, alkylated, and digested with trypsin. The peptide mixture obtained was then fractionated using two different SCX systems, a spin column and a cartridge system, and the fractions were sequentially eluted using 100, 200, 350, and 500 mM KCl. Each fraction was then separately analyzed using LC-MS/MS. The protein-identification results are shown in Table 1. The results from four SCX fractions were compared, revealing 1,421 and 1,590 unique proteins from the spin SCX and cartridge SCX, respectively. The two sets of results were compared, which showed that 1108 proteins were identified in both SCX systems. Because we plan to use only one SCX system in the iTRAQ workflow, only the SCX cartridge system was used in the subsequent labeling experiments. The cartridge SCX approach was compared with the MARS FT approach, which revealed only a small degree of overlap in the identification results. Only 399 proteins were identified in both experiments, and 1,191 and 714 proteins were unique to the SCX and MARS approaches, respectively (Fig. 2). Both sets of results identified 2,304 different proteins.

Considering the widespread use of plasma depletion with affinity fractionation, it appears to be the "gold standard". However, a few other aspects of the MARS approach should be considered. First, the cost of affinity kits and accessories is relatively high (about 4000$ designed for 100 samples), particularly compared to SCX columns (about 400$ for 100 samples). In addition, the SCX pretreatment is about 30% faster than MARS depletion. For instance, after MARS depletion, the samples must be purified through buffer exchange to remove interfering substances. This step is time consuming. After SCX chromatography, the proteins are eluted from the column using increasing KCl concentrations, and the samples are then nearly ready for injection into the LC. An RP C18 pre-column can remove salts from the sample, and only a short evaporation is necessary to remove the ACN from the elution buffer. Indeed, time needed for SCX fractionation is only about 15 minutes, whereas approximately 3-4 hours are necessary for MARS handling. Other steps such as reduction, alkylation and trypsin digestion are the same for both approaches. However, the most important is that the SCX approach is simpler and requires less sample manipulation than the affinity-depletion approach. Another limitation of the affinity kit is the low plasma loading capacity (only 8–10 µl). In fact, the maximum protein quantity that can be obtained after MARS depletion is only ∼50 µg, which may limit further fractionation steps. Because the MARS column is recyclable, one option is to repeat the depletion numerous times and pool the depleted fractions. However, this process is time consuming and requires more sample handling. A multi-stage sample pretreatment using the MARS platform may result in the loss of many proteins, especially during desalting through centrifugal filters, and may be associated with an increased contamination risk. The MARS experiments exhibited the lowest level of reproducibility, which supports this hypothesis.

The iTRAQ reagents are expensive, but compared with other tags, such as TMT and ICAT, the cost of the experiment is similar (∼80$ per sample). Relative quantitation using label-free methods is much cheaper (∼2$ per sample) but more time consuming. In label-free quantitation strategies, each sample must be analyzed separately and with many replicates for a high level of reproducibility. This method cannot be multiplexed and is less precise [33]. Quantitation using iTRAQ labeling facilitates the pooling of samples that belong to the same treatment group, such as treated or untreated. Finally, the cost and time required to prepare and process samples for iTRAQ labeling is relatively low (for example 8–16$ if pooling of 5–10 samples is taken into account). On this basis, we conclude that pretreatment with SCX coupled to iTRAQ labeling and MALDI-TOF/TOF is a simple and inexpensive approach. Based on the high number of proteins identified, this method is also effective and reproducible.

Another aspect must be considered. Off-line nano-LC-MALDI-TOF/TOF is an effective system for protein identification. Nano-LC fractionation directly on a MALDI sample plate decreases the spot complexity and facilitates time-independent peptide analysis because the fractions are “frozen” on the MALDI plate, which facilitates the MS/MS experiments and, thus, longer data acquisition. This method enables the fragmentation of more peptides. Moreover, the increased number of compounds in the MS/MS analyses did not result in the loss of other compounds, in contrast to on-line measurements, in which certain information was lost. Additionally, high-quality fragmentation spectra that are rich in peptide fragment ions facilitate the identification of a high number of proteins. Furthermore, samples may be re-analyzed later under different parameters to optimize the analysis conditions [34]. The only major limitation of the MALDI-based technique compared with the on-line technique is the longer analysis time. Therefore, a more rapid sample pretreatment prior to MS analysis seems to be extremely important.

### Plasma protein quantification using iTRAQ labeling for selected pre-fractionation approaches

**Figure 4:**
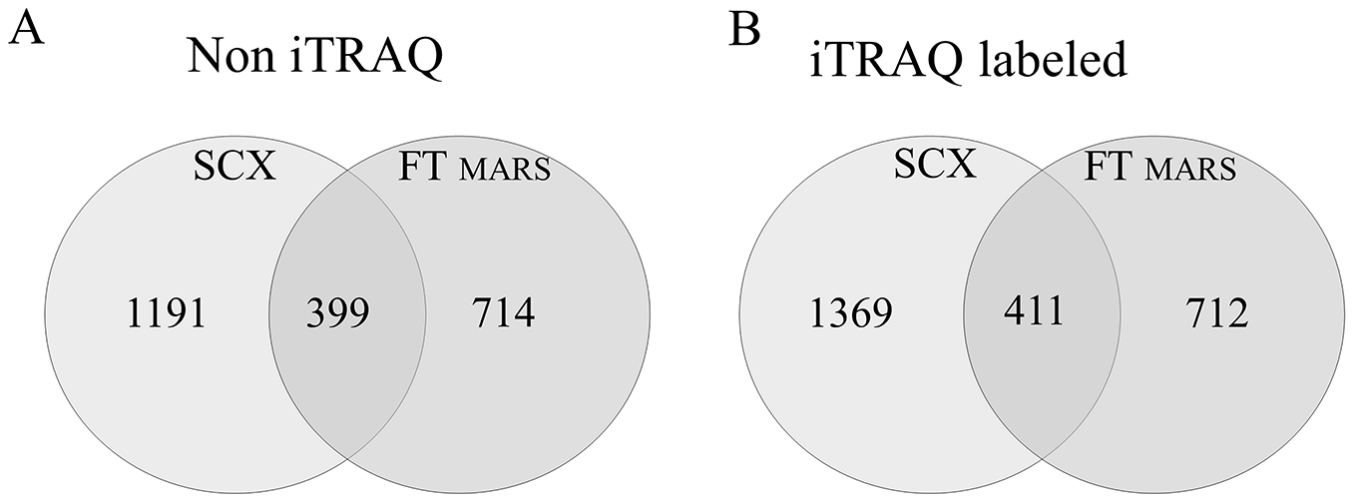
A Venn diagram comparing the results from the MARS FT versus SCX approaches in two experiments: (A) without and (B) with iTRAQ labeling. (A) A total of 2,304 unique proteins were identified: 1,905 proteins were identified using only one method, and 399 proteins were identified by both methods. (B) A total of 2,492 proteins were identified: 2081 of these proteins were identified using only one method, and 411 were identified by both methods.

For iTRAQ quantitation, we selected the technique that yielded the greatest number of high-quality identified proteins. The more peptides identified per protein, the higher the quality and reliability of the results. In the unlabeled experiment, the highest number of proteins was identified using the SCX approach. The MARS FT and WF approaches identified a similar number of proteins (1,113 and 1,098, respectively). However, a qualitative analysis of the data showed that the MARS FT approach yielded better results (i.e., a higher number of unique peptides identified). Using this technique, 276 proteins were identified through 3 or more unique peptides (24.8% of all proteins). In the SCX approach, 251 (15.7%) proteins were identified through 3 or more unique peptides, whereas in the WF approach, only 148 proteins (13.4%) were identified through 3 or more unique peptides (Table 1). On this basis, the SCX and MARS FT approaches were selected for iTRAQ quantitation. However, because the standard iTRAQ/LC-MS approach requires SCX cleaning, both types of samples were subjected to SCX fractionation. Using the MARS FT and SCX pretreatment, we used plasma samples with modifications at quantities required for iTRAQ analysis, which were prepared in accordance with the iTRAQ manual. AB Sciex typically recommends that 50–100 µg of protein be used as the starting material for iTRAQ chemical reactions. To evaluate the labeling efficiency, effectiveness, and accuracy, as well as to mimic real conditions, the samples were labeled with two different tags at the ratio 1∶2. Thus, we labeled peptides derived from 50 µg of protein with the 114 (MARS FT) or 115 (SCX) tags and 100 µg of protein with the 117 tag (MARS FT and SCX) (Table 2). After labeling through both approaches, the samples were purified and fractionated using an SCX cartridge. The peptides were sequentially eluted from the column using 100, 200, 350, and 500 mM KCl. Each fraction was analyzed separately, and the data were compiled. We identified 1,780 and 1,123 unique proteins using the SCX and MARS approaches with iTRAQ labeling, respectively (Tables 1 and 3). Despite the additional fractionation step for MARS FT (a SCX step), these results are similar to the non-iTRAQ experiment (without the additional SCX step for the MARS depleted samples). In an unlabeled approach, 1,590 and 1,113 unique proteins were identified from the SCX and MARS approaches, respectively (Figure 4a). These results suggest that during MARS preparation, protein loss was observed, a problem that was previously reported in other papers; Brands et al. [35] showed that the recovery from MARS affinity separations was approximately 75% on average, but for certain markers, it was approximately 40%. Further, in our practice, we observed that protein affinity depletion leads to the loss of important proteins (data not shown). SCX fractionation also reduces the total number of peptides in the sample by approximately 10–25% [36], [37]; however, the probability that not all peptides for a given protein are lost remains high, and the possibility of protein identification is not excluded. In total, 1,369 and 712 proteins were unique to the SCX and MARS FT approaches in the iTRAQ experiment, respectively, and only 411 proteins were common to both data sets (Figure 4b). The results from both pretreatment methods were compiled from the iTRAQ experiments, wherein 2,492 different proteins were identified (see Table S2 in File S1). The results from both pretreatment methods were also compiled from the unlabeled experiments, wherein 2,304 different proteins were identified. The results obtained for the non-iTRAQ- and iTRAQ-labeled samples are compared in Figure 4.

**Table 2:** iTRAQ-labeling design.

Approach	Isobaric tag	Amount of proteins (µg)
MARS	114	50
MARS	117	100
SCX	115	50
SCX	117	100

To evaluate quantitative accuracy, the samples were labeled with two different tags at the ratio 1:2 of protein amounts.

**Table 3:** Results of iTRAQ labeling for the MARS FT and SCX approaches.

iTRAQ experiment	Total number of non-redundant proteins	No. of quantified proteins (labeled with both iTRAQ tags)	No. of proteins labeled with only the 117 iTRAQ tag
MARS FT	1123±269	863±116	1066±120
SCX	1780±141	1470±99	1707±103

The labeling efficiency for both iTRAQ tags was 76.8% and 82.6% for the MARS FT and SCX approaches, respectively.

In conclusion, the MS-based method that we developed is a robust platform for analyzing and identifying potential novel biomarkers. Using iTRAQ labeling with effective sample pretreatment facilitates the identification and quantification of numerous proteins. Based on the results, we conclude that the SCX approach is the best fractionation technique for plasma protein analysis and for protein identification and comparison. This method yielded the highest number of identified proteins in the labeled and non-labeled experimental sets (Table 3 and Figure 4) and showed a high reproducibility in the number and composition of identified proteins. The lower cost and preparation time compared with the MARS approach are additional advantages. Further, the SCX approach was the only method that allowed plasma fractionation at the peptide level and after iTRAQ labeling. Each fractionation at the protein level increases the number of samples and thus adds to the digestion and labeling cost.

In many studies, SCX fractionation is routinely used after the iTRAQ labeling of affinity-depleted plasma before MS analysis [38]–[41]. Furthermore, an iTRAQ reagent manufacturer (AB Sciex) recommends an SCX step before LC-MS/MS analysis. Ethanol, SDS, triethylammonium bicarbonate, or excess iTRAQ reagents in the labeling protocol may interfere with the LC-MS/MS analysis. Therefore, reducing the concentration of these substances prior to analysis is necessary. The SCX fractionation of liver samples at the protein level has been described [42]. However, to the best of our knowledge, this paper is the first report of SCX fractionation without previous affinity depletion and the first comparison of this approach to MARS depletion in plasma proteome analysis. A high number of unique proteins, a total of 1,123, were also identified using the MARS FT pre-fractionation approach with iTRAQ labeling. Therefore, our results suggest that this technique has a degree of specificity and produces a sample enriched in a specific pool of proteins.

Several studies using iTRAQ plasma protein labeling have been published [38], [43], [44]. Chong et al. identified 174 plasma proteins, although the depletion of high-abundance proteins was performed using a MARS-Hu7 affinity column [38]. Bortner et al. identified 120 and 131 plasma proteins in two different sets of iTRAQ experiments after MARS-Hu14 depletion column treatment [43], and Wiederin identified 220 plasma proteins [44]. Two different depletion methods, an immunodepletion strategy using an IgY-12 spin column and a method using peptide-ligand library technology as well as a ProteoMiner column, were used by Ye et al., who identified 320 proteins in immunodepleted plasma and 248 proteins in hexapeptide ligand library-treated plasma. Only 140 proteins were identified by both methods [39]. Additional studies used immunodepletion of 12 highly abundant serum proteins and identified 160 different proteins [19]. Song et al. have used the same depletion method. Among the 105 identified proteins, only 73 were quantitated in all 8 iTRAQ runs [45]. Two depletion methods and OFFGEL separation were used in another study. In total, 332 proteins from immunodepleted plasma and 320 proteins from hexapeptide-ligand-library-treated plasma were identified [20]. Thus, most methods using iTRAQ labeling identified no more than 250 proteins from plasma [17], [36]–[37], [41]–[43]. An increase in the number of identified proteins was observed in relation to using special equipment for protein separation (e.g., the OFFGEL fractionator) [20]. However, this increase was also associated with increased cost and time. In our study, 1,113 and 1,123 plasma proteins were identified after the MARS-Hu7 depletion method in the non-labeled and iTRAQ-labeled samples, respectively. Better results may be obtained through applying comprehensive, high-resolution, and multi-stage methods for protein pretreatment and fractionation. For example, Faca et al. used a multi-dimensional protein separation system consisting of affinity depletion, anion-exchange, and RP chromatography followed by SDS gel to identify 2,254 plasma proteins [46]. Tang et al. identified 2,890 proteins through a similar approach [47]. Undoubtedly, more intensive fractionation is ideal for reducing sample complexity and may provide an increased number of identified and differentially expressed proteins. Although these multi-stage methods for protein fractionation allowed comprehensive plasma profiling, protein fractionation was not feasible in this study because the iTRAQ labeling was performed at the peptide level. Moreover, our goal was to develop a simple, fast, and inexpensive pretreatment approach that could be widely applied for biomarker discovery using iTRAQ-based proteomics.

**Figure 5:**
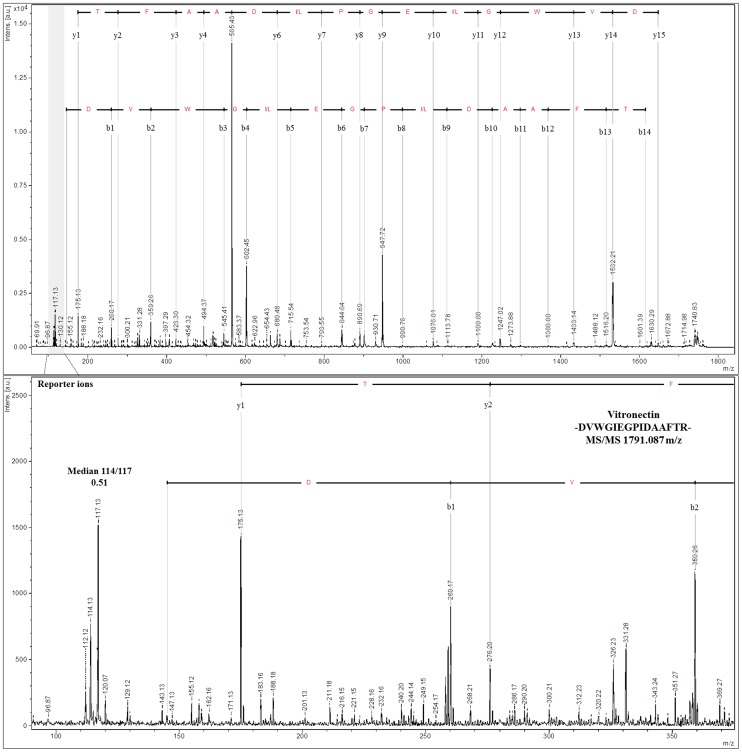
Representative tandem MS spectra demonstrate the range and ratio of the relative intensities observed for the iTRAQ reporter ions using the DVWGIEGPIDAAFTR peptide, which is unique to vitronectin (m/z 1,791.087) (upper panel). The peptides were quantified using the areas of the two iTRAQ reporter ions m/z 114 and m/z 117 (zoom on lower panel). The m/z 114 reporter ion is used for 50-µg samples, and the m/z 117 reporter ion is used for 100-µg plasma samples.

**Figure 6:**
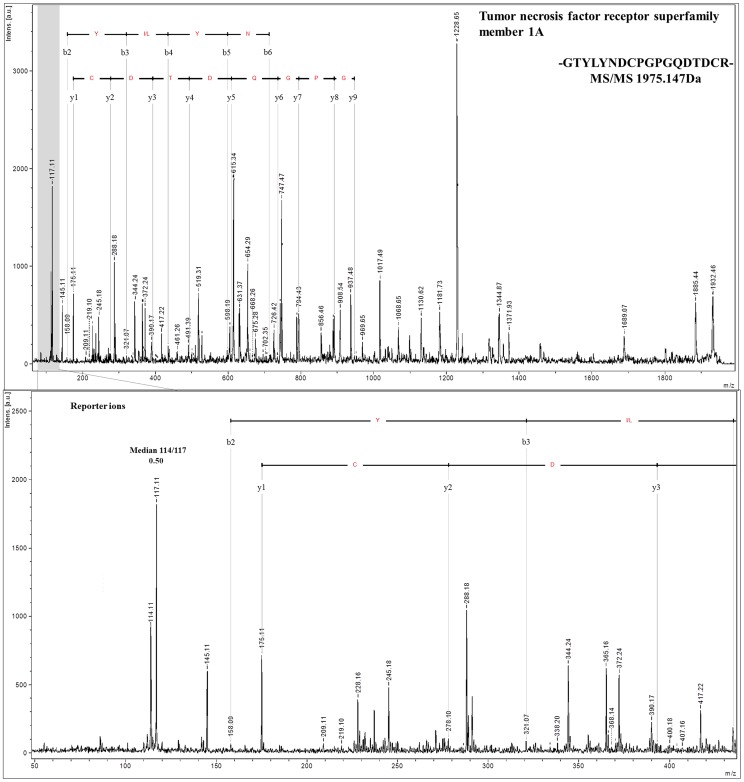
MS/MS spectra for a peptide (m/z 1,795.147) from a low abundance protein, tumor necrosis factor receptor superfamily member 1A, estimated at 35 pg/ml by Polanski et al. 2007. The lower panel zooms in on the iTRAQ reporter ions and correct median values calculated from the peak area ratios.

In the next step, we examined the labeling efficiency and accuracy of iTRAQ to assess the quantitation quality. The reporter ion ratios were automatically calculated from the raw data using the WARP LC software to calculate the MALDI peak areas. Because the samples were labeled using two different tags at the ratio 1∶2, we expected that the fold-change in labeled proteins would be 0.5 (m/z 114/117 or m/z 115/117 peak area ratios). In total, 863 proteins from the MARS FT approach were quantified with an average fold-change of 0.55±0.1 (114/117 tags). More proteins (1,470) were quantified using the SCX approach with an average fold-change of 0.52±0.094 (m/z 115/117 tags). These results indicate that the observed quantification is highly accurate. The labeling efficiency was 76.8% and 82.6% for the MARS FT and SCX approaches, respectively. Manual analysis of the spectra revealed that in almost all spectra, the m/z 117 tags were present (94.9% for MARS FT and 95.9% for SCX out of 1,066 and 1,707 proteins from the MARS FT and SCX approaches, respectively) (Table 3). The representative MS/MS spectra for the reporter ions are shown in Figure 5 and 6. The MS/MS spectra quality in the iTRAQ reporter ion range is a limiting factor for accurate quantitation. The quantification precision generally depends on the signal-to-noise ratio in the MS/MS spectra, and the interference of different precursors during MS precursor selection is important [7]. However, obtaining a quantitative estimate of up- or down-regulated proteins at higher ratios under such conditions is difficult [48]. Kolla et al. used ProteoMiner technology for plasma depletion and identified 235 plasma proteins. However, 187 of the 235 proteins (78.5%) were relatively quantified by analyzing two or more peptides, whereas for 45 proteins, the quantitation was based on a single peptide [18]. Song et al. used serum with immunodepletion of 12 highly abundant proteins and identified 105 proteins, but only 73 presented proteins were quantitated in all eight iTRAQ runs [43]. Moreover, in many studies, the iTRAQ labeling efficiency was not provided; only information on the exclusion of peptides due to the absence of one or more reporter ions from the analysis was shown. On this basis, we conclude that, due to good sample preparation prior to analysis, the results from this work are characterized by high iTRAQ-labeling efficiency

### Qualitative analysis of the results

By combining the iTRAQ-labeled MARS FT and SCX pretreated data sets, 2,492 non-redundant proteins were identified (Table 1; Table S2 in File S1). With the MARS FT approach, 135 (12%) proteins were identified through 3 unique peptides or more, 259 (23%) were identified through 2 peptides, and 788 (65%) were single-peptide proteins. For the SCX approach, 199 (11.2%) proteins were identified through 3 unique peptides or more, 405 (22.7%) were identified through 2 peptides, and 1,176 (66.1%) were single-peptide proteins (Table 1). Because these single-peptide proteins were identified in four separate experiments, these proteins were most likely identified correctly. For the number of proteins identified, the SCX approach produced better results than the MARS approach. All identified proteins were searched in the plasma proteome database available on the web at http://www.plasmaproteomedatabase.org [22]. Forty-eight of the identified proteins were not in this database (see Table S3 in File S1). Forty-two were identified using the SCX approach, whereas only 13 were identified using the MARS FT. These proteins were examined more closely. Three were identified as C-X-C chemokine receptor type 7 and interleukin-22 receptor subunits alpha-1 and alpha-2. C-X-C chemokine receptor type 7 is involved in the tumorogenesis process and could become an important target for new anti-metastatic and anti-cancer drugs [49]. Interleukin 22 receptor-alpha 1 is a member of the class II cytokine receptor family, the members of which are often postulated as potential biomarkers for cancerogenesis and tumor progression [50]. Example MS/MS spectra for proteins not present in the analyzed database are in File S2.

Among the proteins identified, we also found molecules described in the plasma proteome database as low-abundance proteins. Many proteins identified by others at pg/ml concentrations were recognized in our experiments. Among these proteins, one peptide from interleukin-1 alpha, which was described by Polanski et al. as concentrated at 3 pg/ml [51], was identified in our samples using the SCX approach. A single peptide from tumor necrosis factor receptor (Fig. 6) and a single peptide from interleukin 12 were estimated at 35 and 77 pg/ml, respectively [51], [52], and were identified in our study after SCX fractionation. The abnormal production or activity of these proteins has been implicated in many human diseases [53]–[55]. Dysregulation of the TNF receptor, which is the main cell surface receptor for TNF [53], is involved in inflammatory disorders and inflammation processes [54]. The abnormal accumulation of interleukin-12 has been shown in patients with many types of cancers, such as gastric and colorectal cancer as well as hepatocellular carcinoma [55]. Thus, using our approach, we detected plasma proteins even at very low concentrations, including certain proteins that are already considered useful biomarkers. Example MS/MS spectra for low abundance proteins are presented in File S3.

## Conclusions

Sample fractionation is an essential step in proteomic analyses. The development of an improved method for iTRAQ plasma analysis is important for biomarker discovery projects. Plasma depletion using affinity fractionation is routinely used, but simpler methods with fewer steps are a better solution that has been overlooked. Using SCX at the peptide level is an attractive, less time-consuming, inexpensive, and convenient approach for plasma quantitative analysis using iTRAQ labeling. This study demonstrated that four pretreatment platforms provide complementary results for protein identification and quantitation. Each method shows particular specificity with unique enrichment of a specific pool of proteins, and little overlap was observed between the proteins identified using these techniques. This phenomenon may explain why it is difficult to obtain reproducible results in different laboratories. By combining these techniques, 3,296 unique proteins were identified. For a single pretreatment, the best results were obtained using the SCX approach, which resulted in the enrichment of low-abundance proteins, which are considered valuable potential biomarker sources. This platform also yielded the greatest number of identified proteins, reproducibility, and labeling efficiency. Using this approach, 1,780 proteins were identified with an 82.6% labeling efficiency. Of these identified proteins, 604 were identified with two or more unique peptides. Based on the results of this study, it may be advantageous to use the SCX system instead of the widely used MARS platform for iTRAQ studies because the SCX method significantly increases the number of identified and quantified protein biomarkers in plasma proteomics experiments.

## Supporting Information

File S1 **This material includes a complete list of the proteins identified in approaches without (Table S1) and with iTRAQ labeling (Table S2).** Further, proteins that were not in the plasma protein database are listed (Table S3). The Tables provide information on the identified proteins (*e.g.*, the SwissProt accession number, MALDI score, sequence coverage, number of identified peptides, MW, pI, relative quantitation of the identified proteins, median values calculated from the reporter peak area ratios, and number of iTRAQ labeled peptides).
(XLS)

File S2 **This supplementary material includes examples of MS/MS spectra of peptides from proteins that were not in the plasma protein database (** http://www.plasmaproteomedatabase.org **).**
(PDF)

File S3 **This supplementary material includes examples of MS/MS spectra of peptides from low-abundance proteins identified in our work and detected by others at pg/ml.**
(PDF)
