# Supplementary material for: Optimization of Plasma Sample Pretreatment for Quantitative Analysis Using iTRAQ Labeling and LC-MALDI-TOF/TOF
Source: PLoS One. 2014 Jul 2;9(7):e101694. doi: 10.1371/journal.pone.0101694 (PMC4079693; doi:10.1371/journal.pone.0101694)
Supplement: File S2 — This supplementary material includes examples of MS/MS spectra of peptides from proteins that were not in the plasma protein database ( http://www.plasmaproteomedatabase.org ). (PDF) [file pone.0101694.s002.pdf]

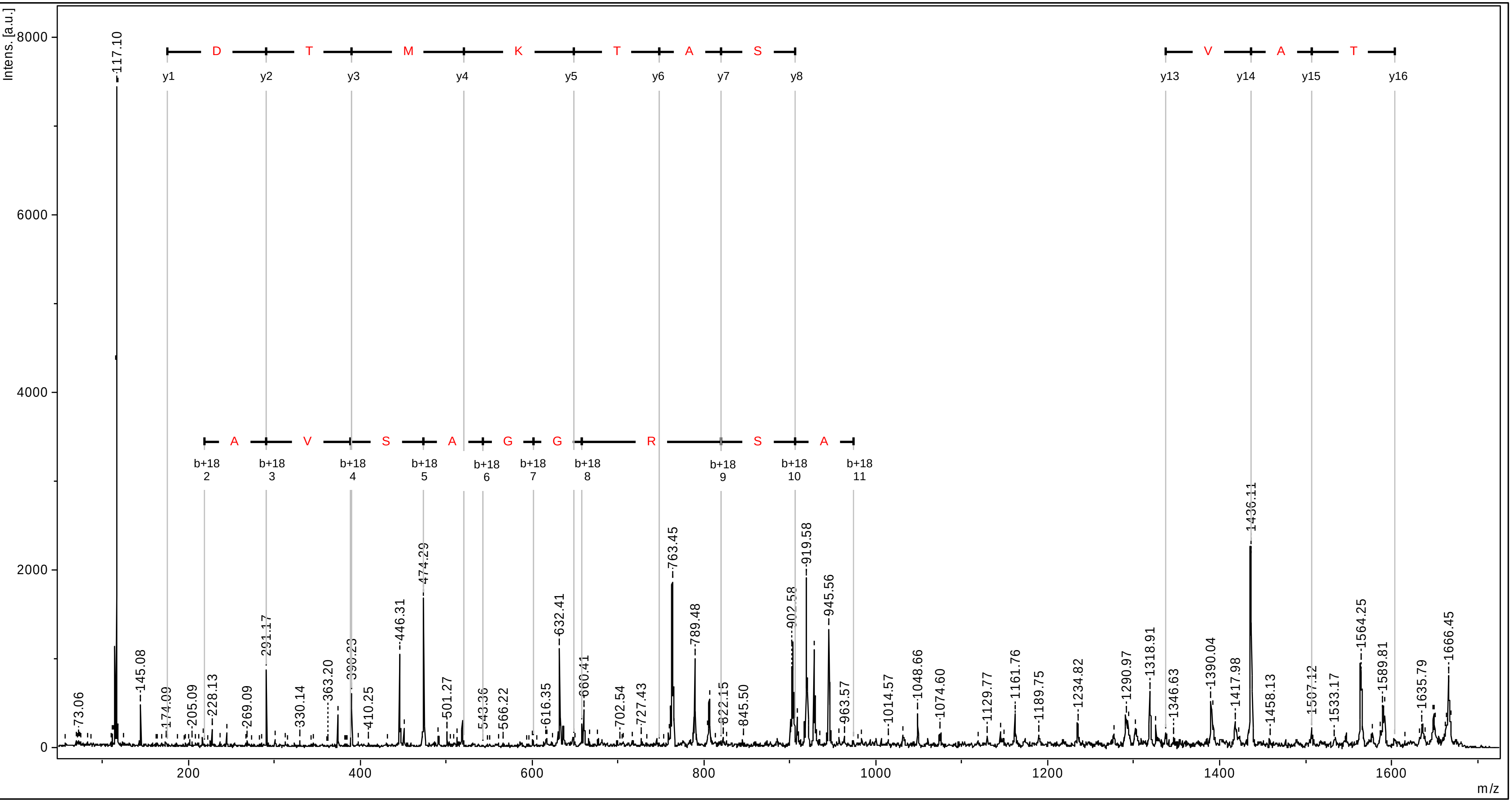

Interleukin-22 receptor subunit alpha-1 IL22RA1

VTAVSAGGRSATKMTDR 1706.061 Da

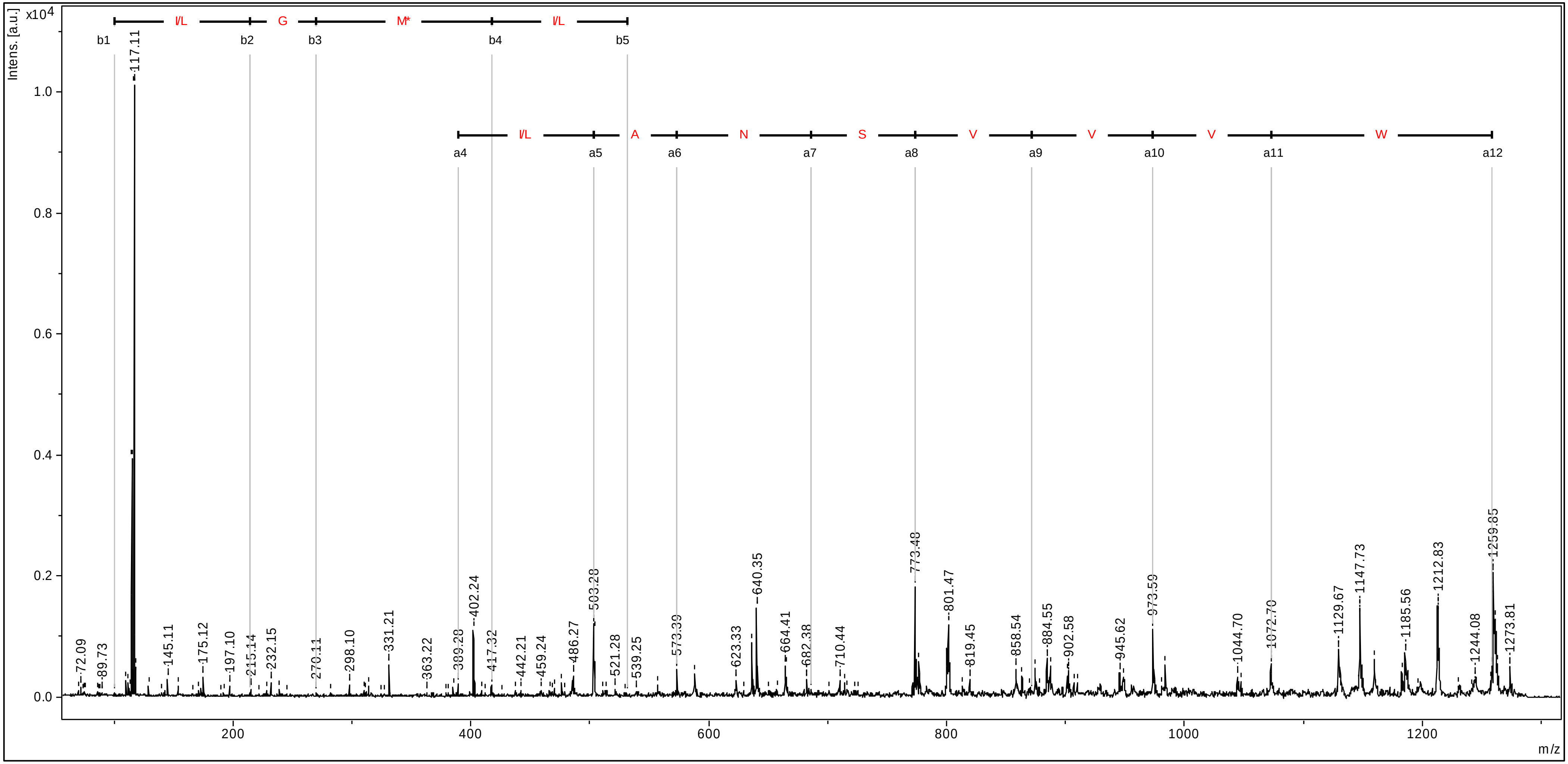

C-X-C chemokine receptor type 7 CXCR7

VIGMIANSVVVW 1303.792 Da

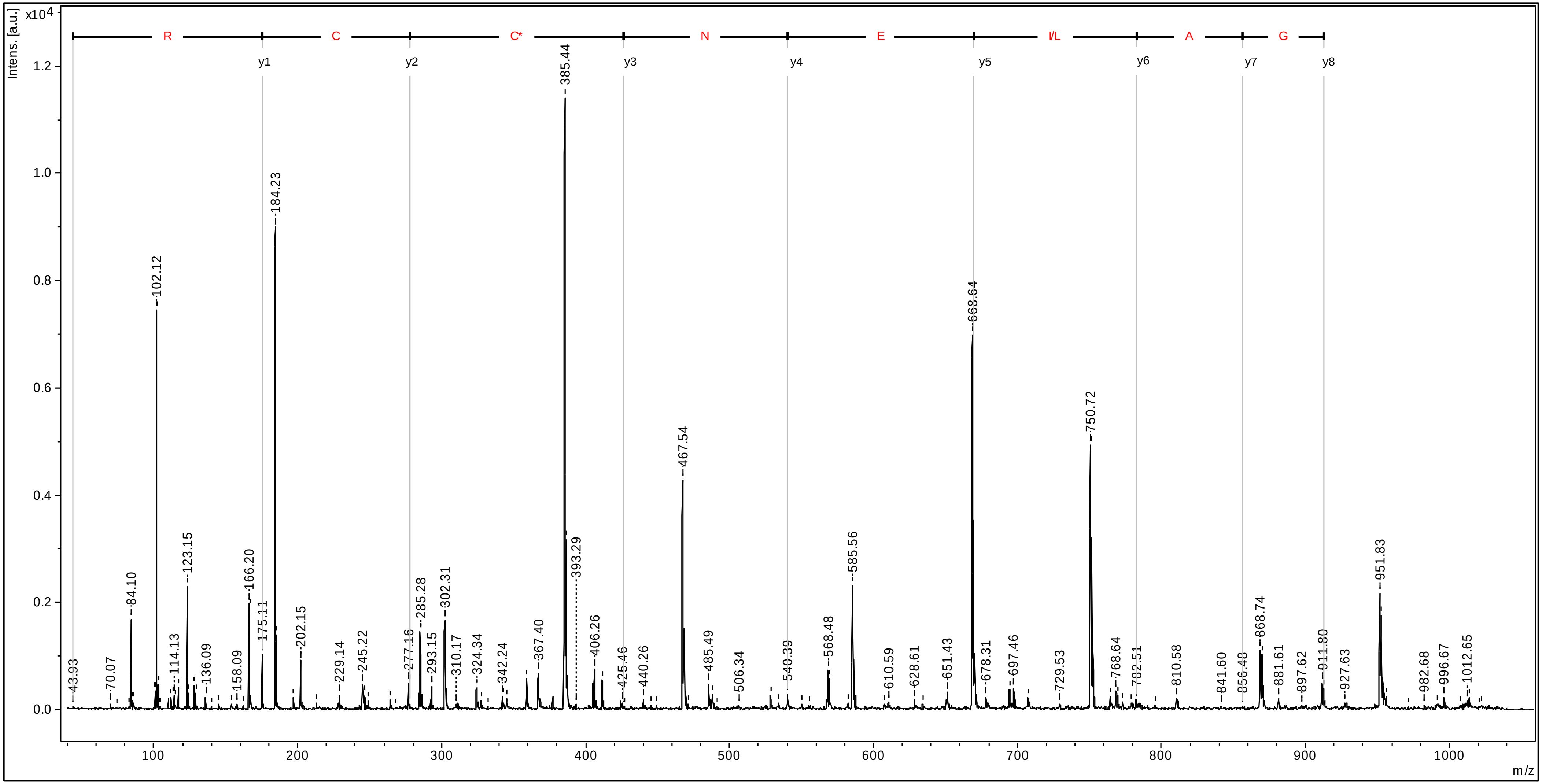

Insulin-like growth factor 2 mRNA-binding protein 1 IGF2BP1

GAIENCCR   1055.455Da

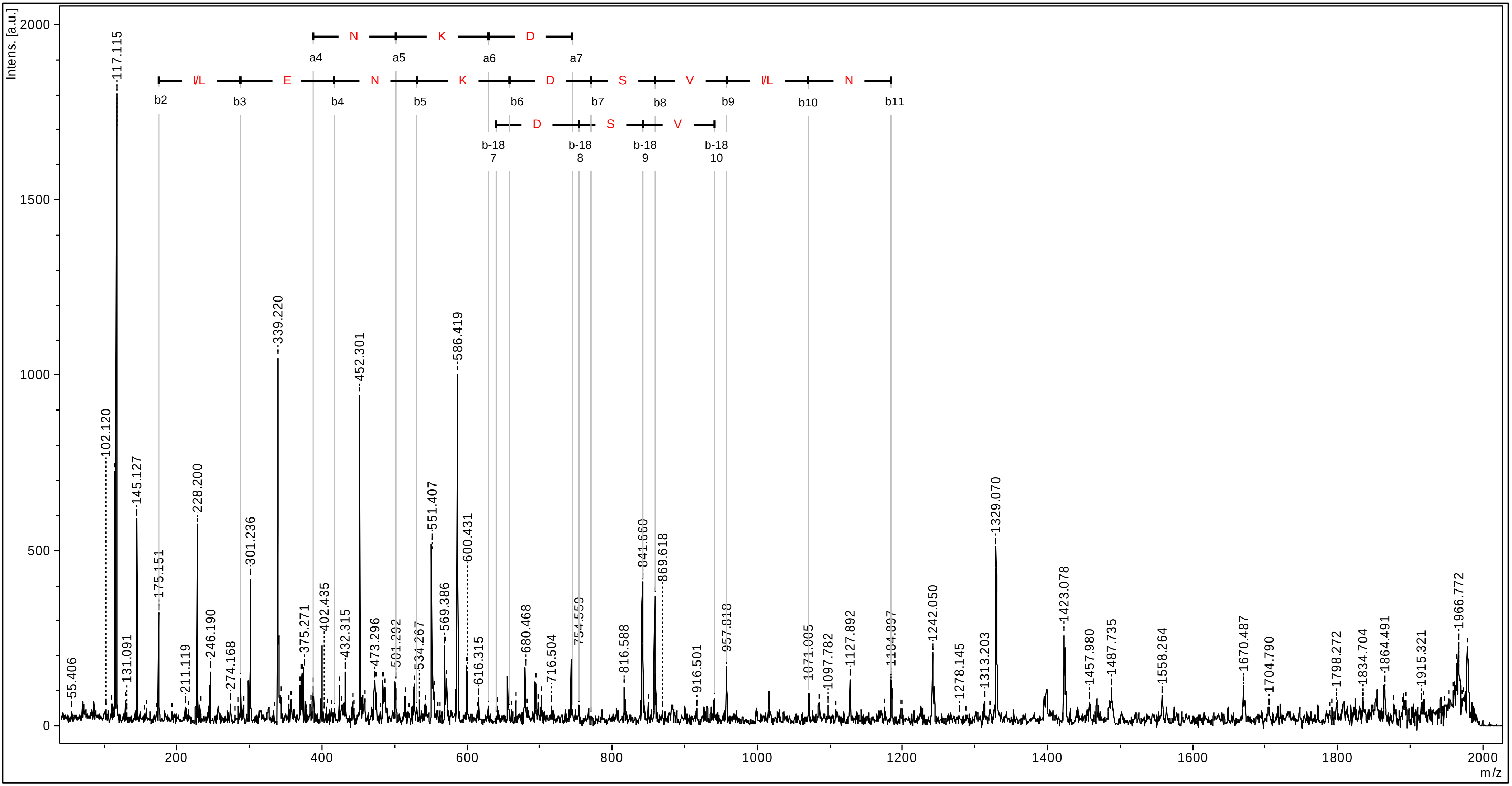

Ankyrin repeat domain-containing protein 36B ANKRD36B

ATIENKDSVLNTATKMK 2008.274 Da

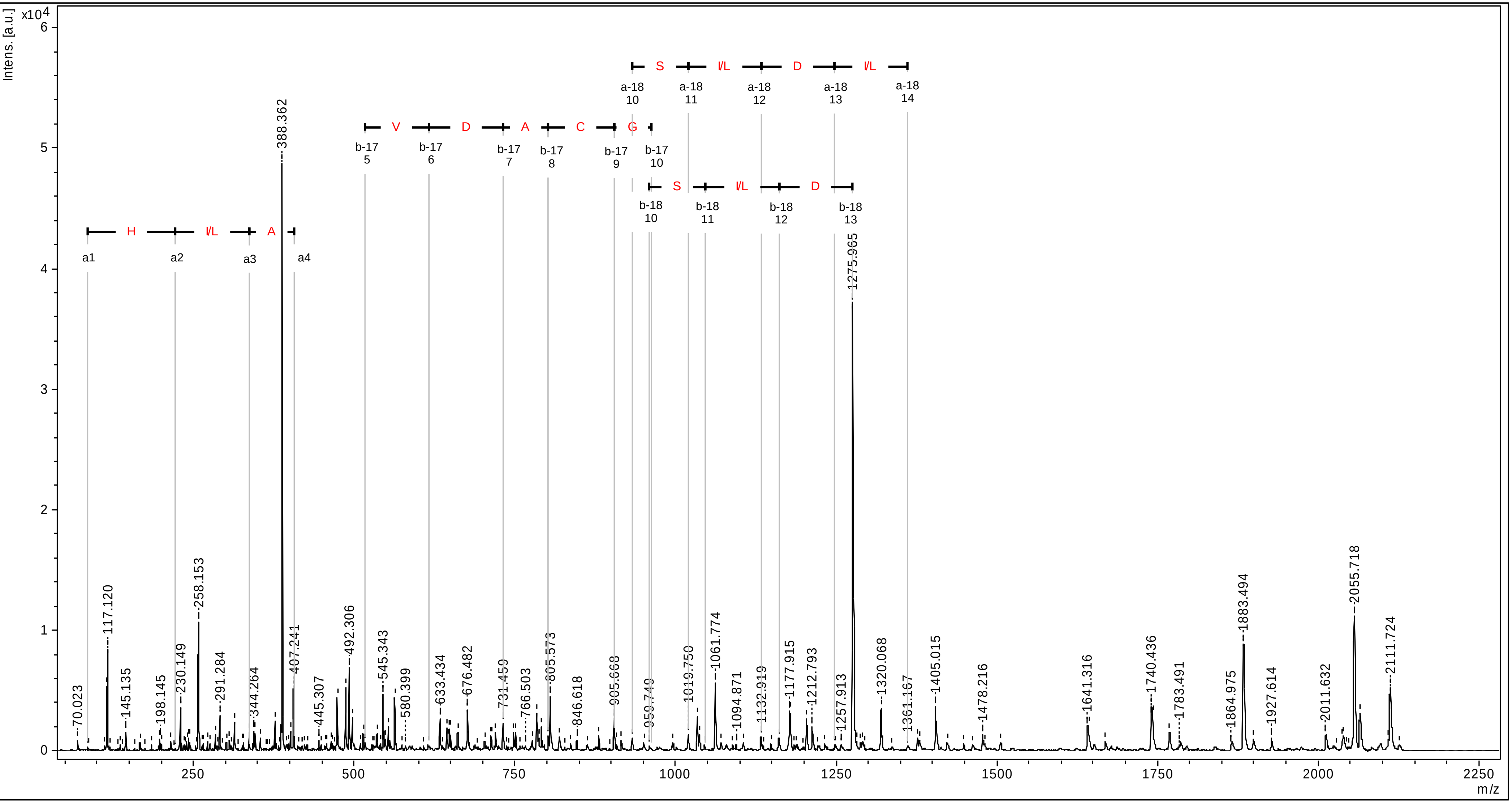

Zinc finger protein 862 ZNF862

LHLAVVDACGSIDLVKKCDR 2155.135 Da
