# Supplementary material for: Optimization of Plasma Sample Pretreatment for Quantitative Analysis Using iTRAQ Labeling and LC-MALDI-TOF/TOF
Source: PLoS One. 2014 Jul 2;9(7):e101694. doi: 10.1371/journal.pone.0101694 (PMC4079693; doi:10.1371/journal.pone.0101694)
Supplement: File S3 — This supplementary material includes examples of MS/MS spectra of peptides from low-abundance proteins identified in our work and detected by others at pg/ml. (PDF) [file pone.0101694.s003.pdf]

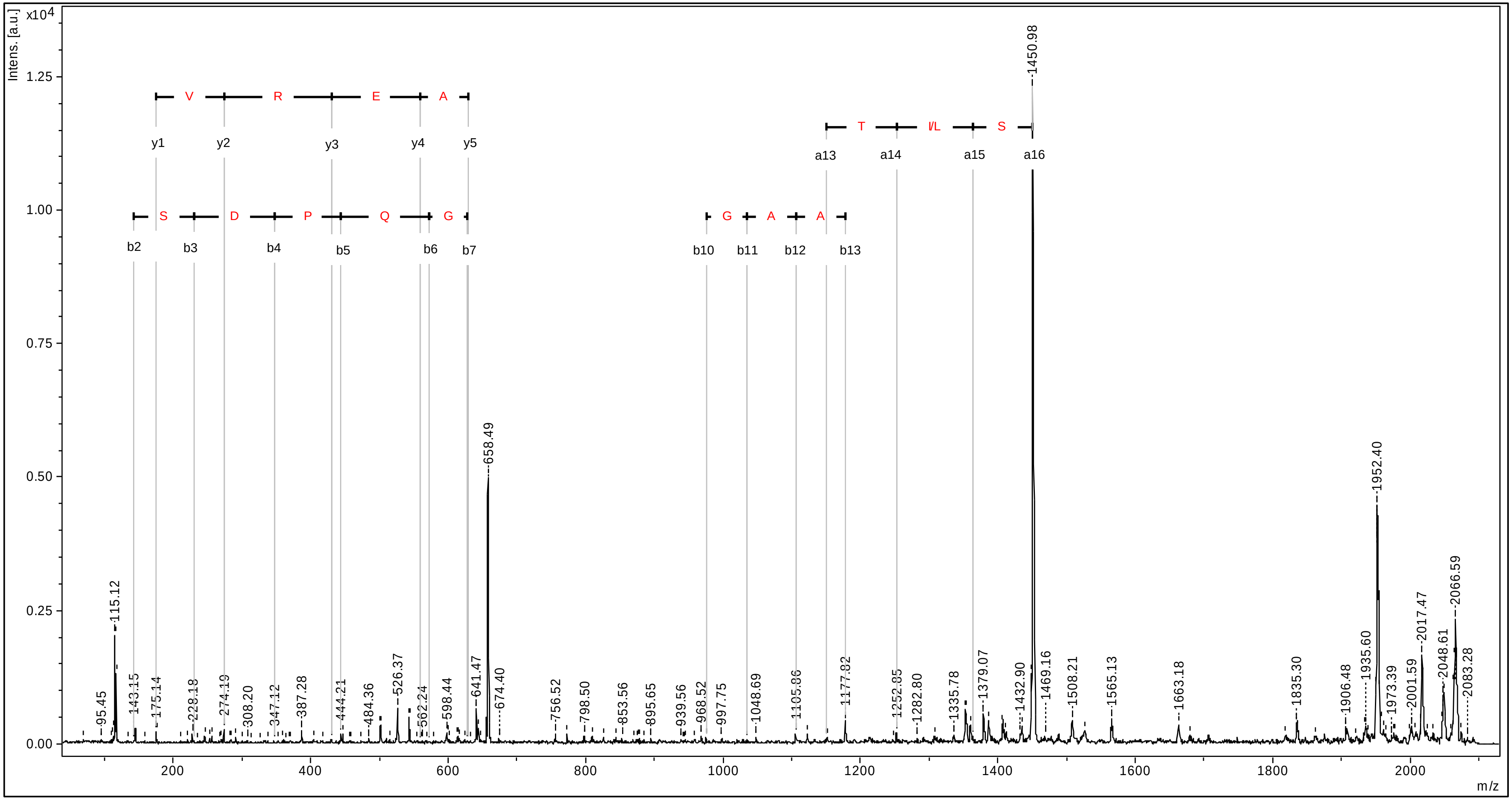

Interleukin 12 subunit beta IL12B

GSSDPQGVTCGAATLSAERVR 2108.157 Da
Protein concentration: 77 pg/ml (Shen et al. 2004)

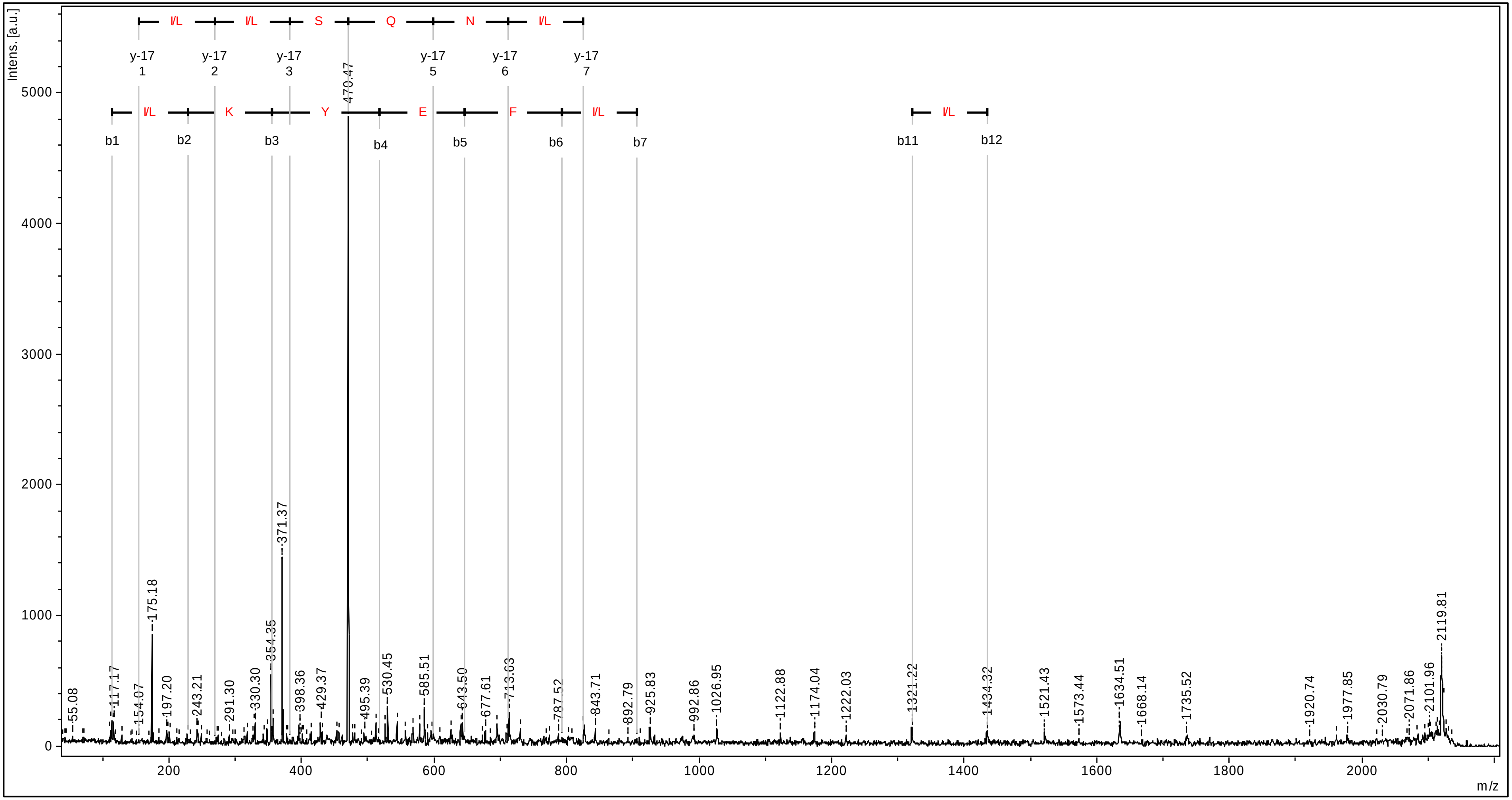

Interleukin-1 alpha IL1A

IIKYEFILNDALNQSIIR 2163.395 Da

Protein concentration: 3 pg/ml (Polanski et al. 2007)

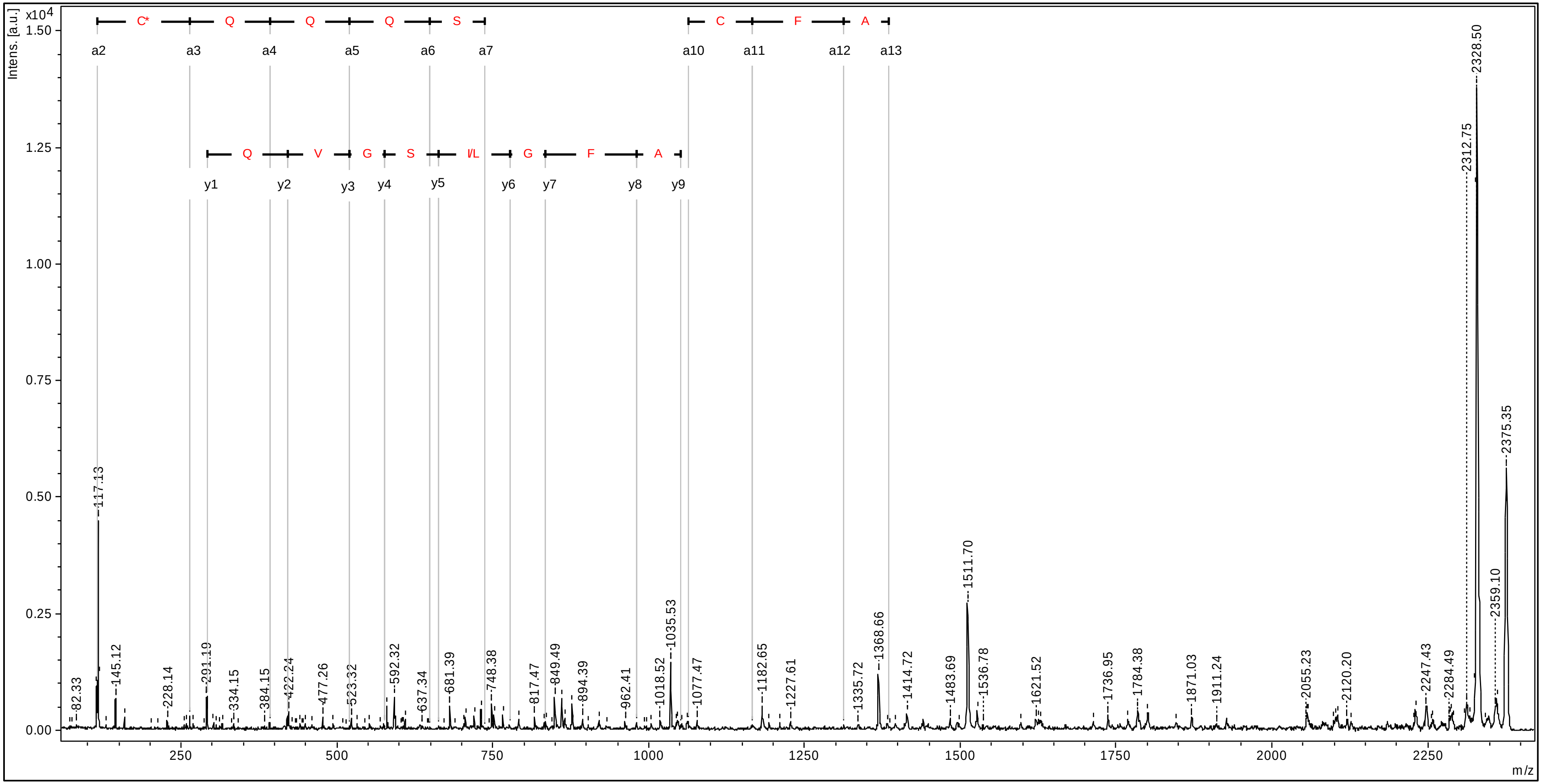

Interleukin-33 IL33

AACQQQSTVECFAFGISGVQK 2392.302

Protein concentration: 95.9+/-236.9 pg/ml (Hong *et al.* 2011)

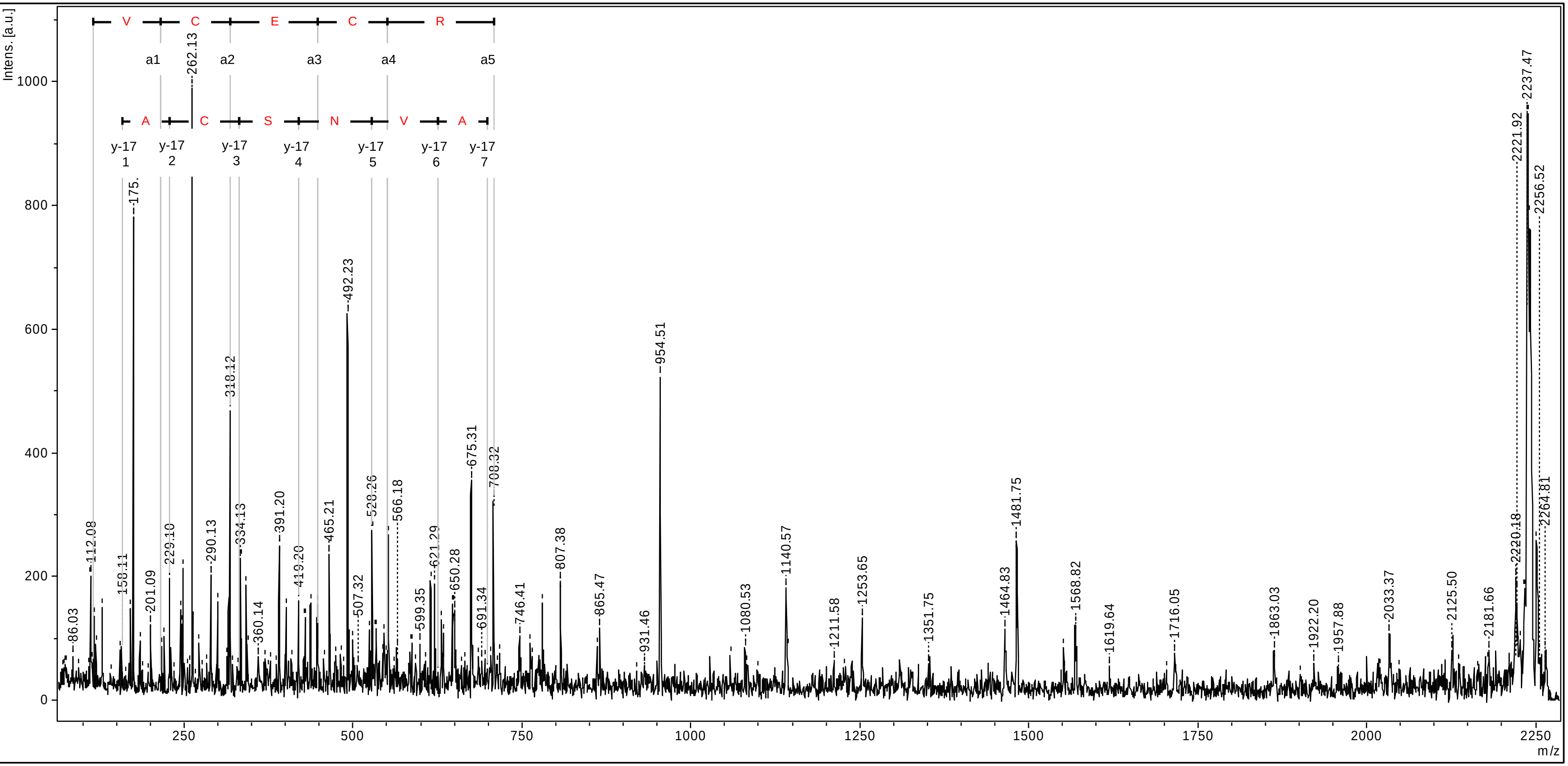

Tumor necrosis factor receptor superfamily member 8 TNFRSF8

VCECRPGMFCSTSAVNSCAR   2281.240 Da

Protein concentration: 330 pg/ml (Haab *et al.* 2005)

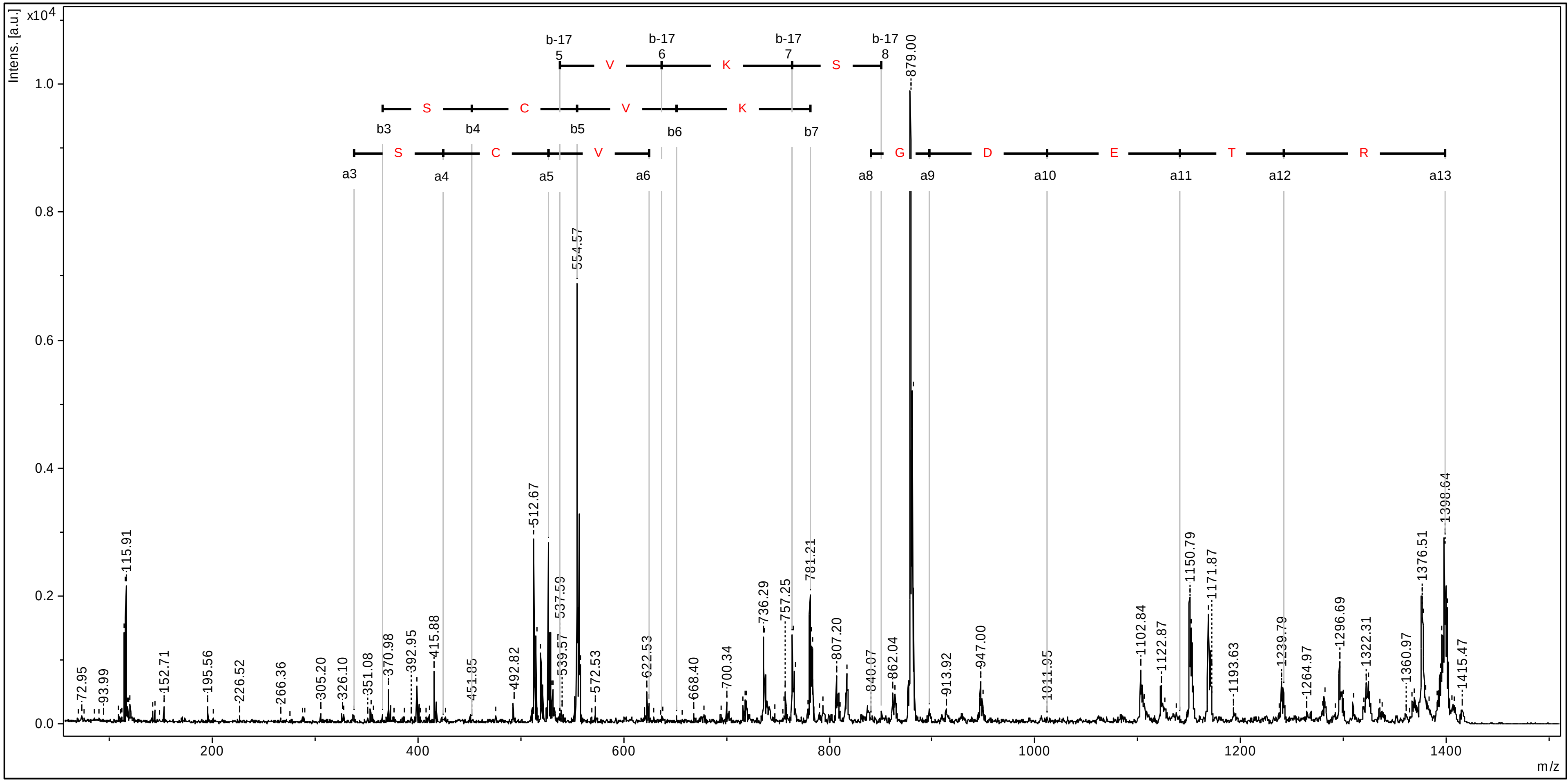

Interleukin-1 receptor antagonist protein IL1RN

MCLSCVKSGDETR 1444.796 Da

Protein concentration: 236 pg/ml (Liu *et al.* 2005)

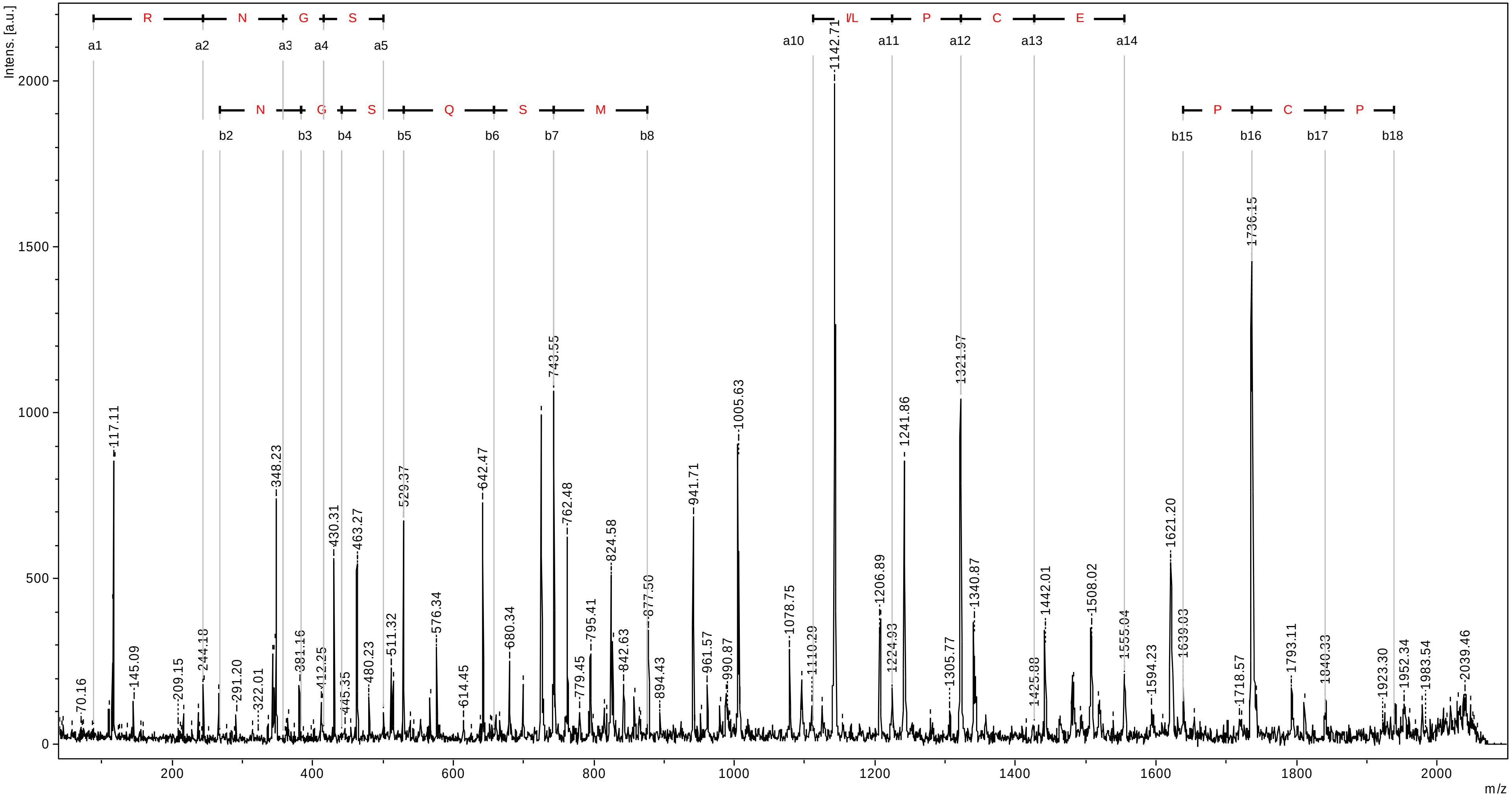

Insulin-like growth factor 1 receptor IGF1R

IRNGSQSMYCIPCEGPCPK 2083.323 Da

Protein concentration: 240 pg/ml (Haab *et al.* 2005)

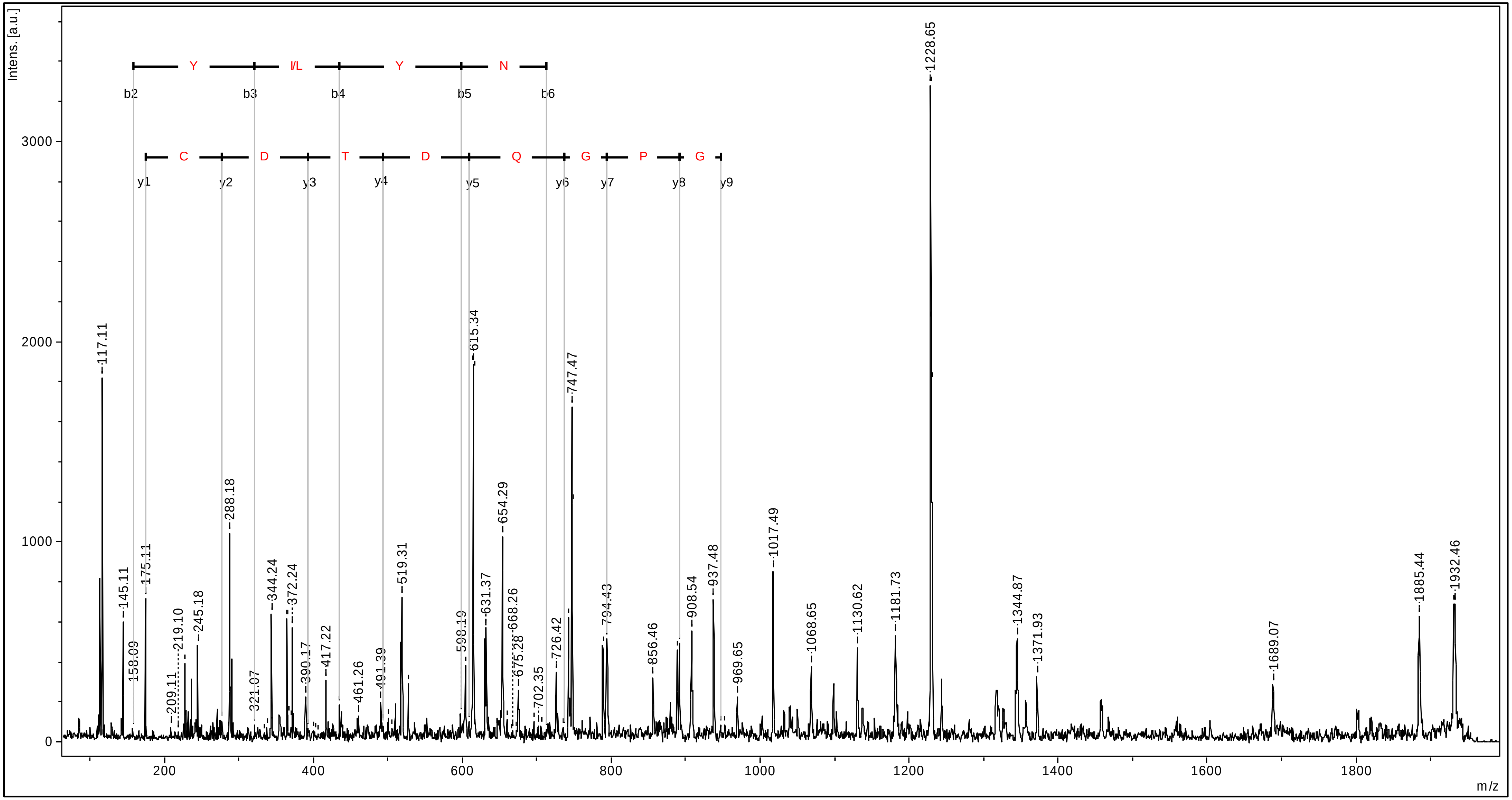

Tumor necrosis factor receptor superfamily member 1A   TNFRSF1A

GTYLYNDCPGPGQDTDCR 1975.147Da

Protein concentration: 35 pg/ml (Polanski *et al.* 2007)
